# A novel protocol of whole mount electro-immunofluorescence staining

**Published:** 2009-03-06

**Authors:** Hongshan Liu, Winston W.Y. Kao

**Affiliations:** 1 Department of Ophthalmology, University of Cincinnati, Cincinnati, OH; 2 Department of Cell Biology and Cancer Biology, University of Cincinnati, Cincinnati, OH

## Abstract

**Purpose:**

To develop a new method of whole mount immunostaining that improves the penetration of staining reagents into the cornea and decreases non-specific binding and background.

**Methods:**

Adult mouse corneas were fixed overnight in 4% paraformaldehyde or a mixture of 4% paraformaldehyde and 0.2% glutaraldehyde in 0.1 M phosphate buffer, pH 7.4, at 4 °C. After washing with 0.1% Triton X-100, corneas were embedded in 1% solidified agarose in a plastic column and fluorescent staining reagents, e.g., FITC-IgG (Fluorescein isothiocyanate- immunoglobulinG) conjugates in 0.5% solidified agarose was overlaid onto the specimens. The column was directionally immersed in a submarine gel electrophoresis apparatus filled with Tris-glycine buffer (TGB, pH=7.4) and electrophoresed at 4–10 mA for 10–24 h. For comparison, conventional protocols of immune fluorescent staining were also employed. The outcomes were evaluated by confocal microscopy.

**Results:**

Antibody conjugates recognizing extracellular matrix (ECM) components, integral membrane protein, and intracellular structural proteins were used in whole mount corneas. The images of confocal laser scanning microscopy (CLSM) displayed a uniform distribution pattern of keratocan in corneal stroma, which is similar to that of section-staining. Anti-β-tubulin antibodies bound to microtubes that are distributed within the whole cell body of superficial corneal epithelium cells and stromal keratocytes, but it was found perinuclear of corneal epithelial wing layers and endothelium; integral membrane protein, FAK (focal adhesion kinase), specifically labeled stromal cells of keratectomy corneas that healed for three weeks. In comparison, conventional protocols of immune fluorescent staining using the same antibody conjugates were also employed but did not yield satisfactory results. It was found that IgG conjugates examined did not readily penetrate into stroma and/or intact corneal epithelium. Phalloidin is a small molecule that can readily penetrate into deep tissue and preferentially binds to F-actin. After the whole mount electrofluorescent staining of phalloidin-rhodamine in the mouse cornea, the results were the same as conventional whole mount staining during the healing of epithelial debridement. The cytoplasmic protrusion formed by lamellipodia and filopodia can be clearly demonstrated.

**Conclusions:**

These results indicate that the whole mount electro-immunofluorescent staining allows the detection of antigens in all layers of cornea, i.e., epithelium, stroma, and endothelium.

## Introduction

Immunohistochemistry is an important technique and is widely used to determine the distribution of gene products in normal and diseased tissues. Many reports have demonstrated that a significant amount of protein is extracted from the tissue during dehydration and embedding procedures employed with ordinary immunohistochemical protocols [1-4]. Thus, it often compromises the evaluation of whether the final immunohistochemical patterns accurately reflect the content and distribution of the proteins in situ. This pitfall can be partially alleviated by using whole mount immunohistochemistry that avoids extensive and laborious procedures of dehydration, clearing, and embedding. The mild tissue processing procedures of whole mount immunostaining allows better preservation of antigenicity in the tissues. Further, the combination of whole mount immunostaining and high resolution confocal laser scanning microscopy (CLSM) provides the capability of using optical sectioning through thick tissue sections (up to 100 μm) to illustrate a three dimensional (3D) structure that accurately displays the distribution of the antigen in situ. However, the inherent difficulty of poor antibody penetration into the thick tissue sections greatly hampers the application of whole mount immunohistochemistry in research and clinical diagnosis [5-8]. Immunoreagents were limited to a penetration depth of 8–9 µm, especially in dense and compact tissue such as brain tissue [9]. To get better penetration of the staining reagents into tissues, incubation times must often be extended for a couple days in conjunction with the use of nonionic detergent, e.g., Triton X-100. Nonetheless, non-specific binding in thick tissues remains a major problem of whole mount immunohistochemistry [1,10-17].

The cornea is a compact and dense tissue and primarily consists of three distinct layers i.e., epithelium, stroma, and endothelium. The corneal endothelium consists of a single layer of hexagonal cells with tight intercellular junctions and forms the posterior barrier of the cornea. The epithelium forms the anterior barrier of the cornea and comprises several layers of stratified epithelial cells connected by tight junctions that greatly limit diffusion of antibody molecules into the tissues. In mouse, the corneal thickness is approximately 120 µm. It is generally experienced that IgG (immunoglobulin G) molecules do not readily penetrate through the epithelium and reach the corneal stroma [14].

The purpose of this study was to develop a protocol using electric current to drive IgG conjugates and other staining reagents, e.g., phalloidin, deep into tissues for determination of antigen distribution and actin stress fiber in situ, respectively.

## Methods

All animal experiments were in compliance with the Statement of Association for Research in Vision and Ophthalmology for the Use of Animals in Ophthalmic and Vision Research, and all procedures were approved by Institutional Animal Care and Use Committee of the University of Cincinnati, Cincinnati, OH.

Affinity purified goat anti-mouse keratocan was conjugated to Alexa 555 using procedures recommended by the manufacturer (Invitrogen/Molecular Probes, Eugene, OR). Phalloidin-rhodamine was purchased from Invitrogen/Molecular Probes. Mouse monoclonal anti-β-tubulin-Alexa 555 and mouse anti-FAK-Alexa 555 as well as normal goat and mouse IgG conjugates were purchased from Upstate Cell Signaling Solutions (Lake Placid, NY).

To determine net electric charge of staining reagents in the buffer, the IgG conjugates were loaded into a 1% agarose gel in Tris-glycine buffer (TGB, pH 7.4), which contained 25 mM Tris and 250 mM glycine/HCl, and then electrophoresed at 10 mA to determine the net charge of IgG conjugates in TGB. After 4–6 h, the gels were examined with a fluorescent stereoscope. If the fluorescent signal migrated toward the positive pole of submarine gel electrophoresis apparatus, the staining reagent tested is negatively charged in Tris-glycine buffer. Otherwise, it is positively charged.

### Tissue preparation

Wild type mice were euthanatized, and the eyeball was fixed in 4% paraformaldehyde or a mixture of 4% paraformaldehyde and 0.2% glutaraldehyde in 0.1 M phosphate buffer, pH 7.4, at 4 °C overnight. The combined fixation with 0.2% glutaraldehyde and 4% paraformaldehyde reduces the loss of soluble cytoplasmic proteins during electrophoresis of antibody conjugates into tissues while preserving antigenicity. We have observed that green fluorescence of tissues expressing EGFP (enhanced green fluorescent protein) remained after electro-immunostaining (our unpublished observation). After removal of the iris, lens, and posterior tissues, the cornea was washed in TGB and incubated with 0.1% Triton X-100 in TGB for 1 h.

### Immunoelectrophoresis

**Figure 1:**
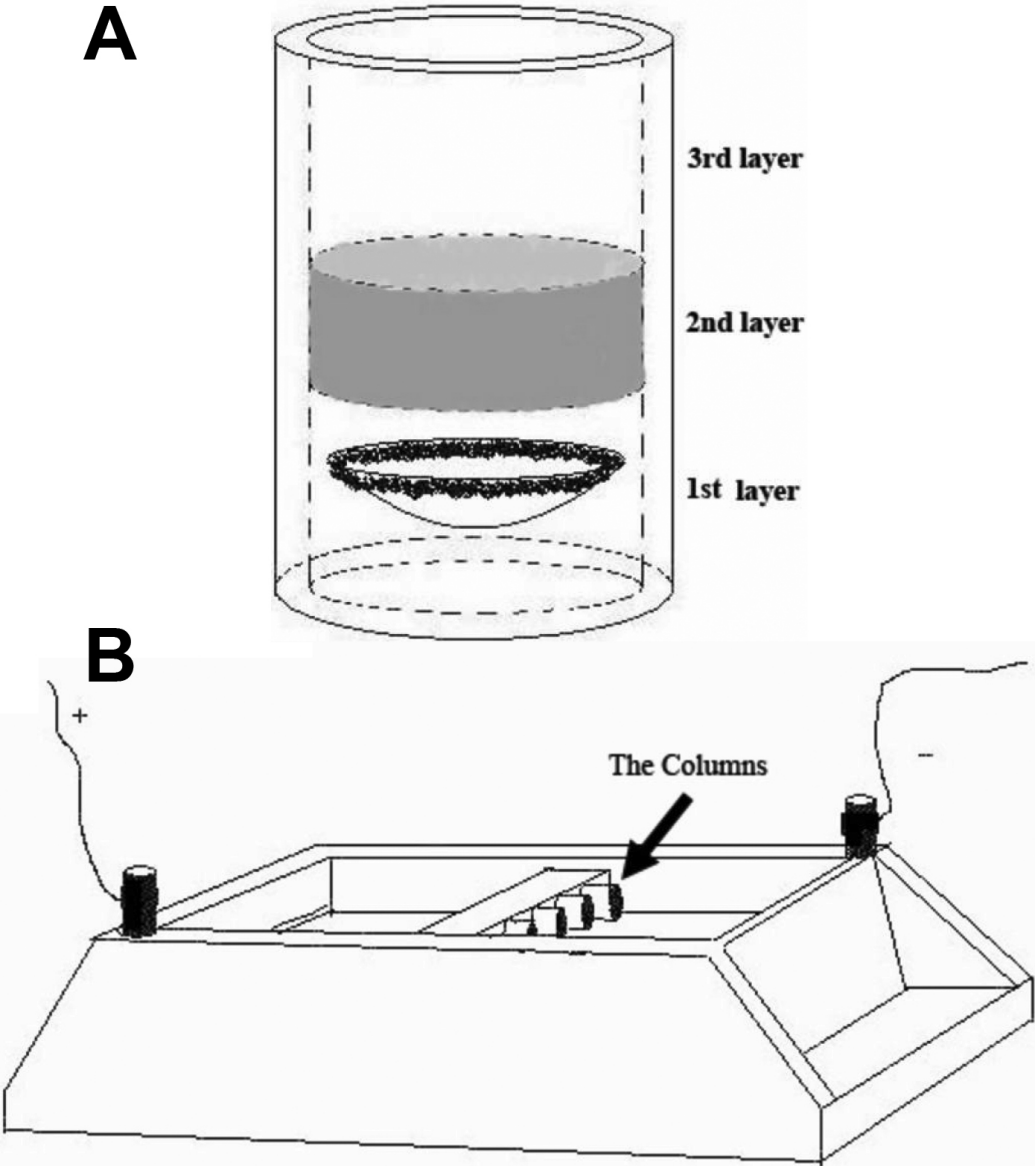
Diagrams of the electrophoresis apparatus. **A**: The column is made of plastic tubing, the inner diameter is about 6 mm, and the length is 15 mm. The column consisted of three layers. The first layer contains a cornea (or other tissue) embedded in 1% agarose in 0.05% Triton X-100 in TGB. The second layer is 0.5% agarose containing IgG fluorescent conjugate in TGB, and the third layer is 2% agarose in TGB. **B**: Depending on predetermined net charges of the reagent in TGB (pH 7.4), the column was directionally immersed in a submarine gel electrophoresis apparatus filled with 1X TGB, and then the IgG conjugates were electrophoresed into the cornea at 4–10 mA.

The bottom of the electrophoresis column was sealed, and the cornea was embedded with endothelium face up in 100 µl of 1% agarose (50–60 °C) in TGB. After the agarose solidified, an aliquot of 100 µl of antibody conjugates in 0.5% agarose (50–60 °C) was overlaid on top of the specimen and then sealed with 100 µl of 2% agarose in TGB (Figure 1A). Depending on the predetermined net charge of the reagent in TGB (pH 7.4) as shown in Table 1, the column was directionally immersed in a submarine gel electrophoresis apparatus filled with 1X TGB, and then the IgG-conjugates were electrophoresed into the cornea for 10–24 h at 4–10 mA (Figure 1B). The specimens were then examined by confocal laser scanning microscopy (CLSM).

**Table 1:** Staining reagents used and their net charge.

**Reagents**	**Net charge**
Goat anti-keratocan C-Alexa 555	Positive
Phalloidin-TRITC	Positive
Mouse anti-β-tubulin-Alexa 555	Negative
Mouse anti-FAK-Alexa 555	Negative
Normal goat IgG-Alexa 555	Negative
Normal mouse IgG-Alexa 555	Negative
DAPI	Positive

The net charge was determined in Tris-glycine/HCl buffer, pH 7.4.

### Conventional fluorescent whole mount staining

For comparison to the novel electro-immunostaining, we performed whole mount staining with protocols described by Grzanna et al. [18] and Burchanowski et al. [19] with the staining reagents phalloidin-rhodamine, anti-keratocan-Alexa555, and anti-β-tubulin-Alexa 555 in normal C57BL/6J mouse corneas as well as the staining reagent anti-FAK-Alexa 555 in C57BL/6J mouse corneas three weeks after keratectomy. Briefly, the cornea was fixed overnight in 4% paraformaldehyde contained in 0.1 M phosphate buffer (pH 7.4) or in the mixture of 4% paraformaldehyde and 0.2% glutaraldehyde, which was also contained in 0.1 M phosphate buffer (pH 7.4), at 4 °C. The cornea was incubated in the staining reagent in PBS containing 0.4% Triton X-100 for 24 h at 4 °C and then was washed in PBS containing 0.02% Triton X-100 for 3 h (the wash was repeated for 10 changes). Finally, the specimen was mounted on the slide and examined by CLSM.

### Corneal epithelial debridement and keratectomy

To examine the feasibility of using whole mount electro-immunostaining to evaluate healing of injured corneas, adult C57BL/6J mice were anesthetized with an IP (intra-peritoneal) injection of xylazine and ketamine as described before [20]. Corneal epithelial debridement and keratectomy were performed with an Agerbrush® (The Alger Equipment Company, Inc., Lago Vista, TX) and beaver blade, respectively. Following the healing of epithelium debridement, which lasted 12–18 h and keratectomy, which lasted for three weeks, the eyeballs were fixed as described above. The cornea was cut down for whole mount electrofluorescent staining with phalloidin-rhodamine conjugate and focal adhesion kinase (FAK), respectively. CLSM was employed for the assessment of corneal epithelial migration.

### Confocal laser scanning microscopy

The cornea was mounted on a slide and imaged using a 40X water-immersion objective lens in two-photon CLSM (Zeiss 510; Carl Zeiss MicroImaging, Inc., Thornwood, NY). The fluorescent signal detection from Alexa 555 and rhodamine was obtained using the 543 nm laser line of the red helium-neon laser. Emitted light was detected using a 565–615 nm filter. DAPI (4',6-diamidino-2-phenylindole) signal was caught using a model-locked MaiTai laser (Carl Zeiss).

## Results

### Net electric charges of staining reagents and electrophoresis time

To determine the migration direction of the staining reagents, we tested net electric charge of the staining reagents, i.e., pre-labeled IgG conjugates and nuclear and cytoskeleton staining dyes in TGB (pH 7.4). Table 1 shows the net charges of staining reagents used in the present study.

### Whole mount electro-immunofluorescent staining for extracellular matrix components

**Figure 2:**
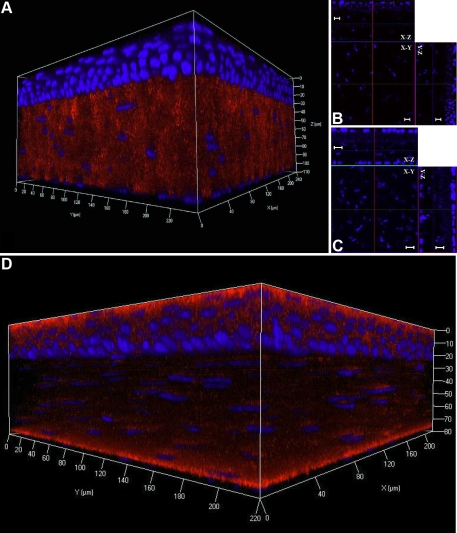
Whole mount electro-immunofluorescent staining with anti-keratocan. Wild type mouse corneal stroma was labeled by goat anti-keratocan Alexa555 conjugate (red) and nuclei were stained with DAPI (blue) as described in Methods. The red fluorescence is only detected in corneal stroma (**A**). No red fluorescent signals are found in the keratocan knockout mouse cornea by goat anti-keratocan Alexa555 conjugate (**B**). The wild type mouse corneal stroma is not labeled by non-immune goat IgG Alexa555 conjugate (**C**). Conventional whole mount fluorescent immunostaining with anti-keratocan-Alexa 555 conjugate showed that the antibody couldn’t penetrated into the deep corneal stroma and exhibited non-specific binding on the surface of the corneal epithelium and endothelium (**D**). Scale bar: 10 μm.

Keratocan is an important extracellular matrix (ECM) component in the stroma for the maintenance of corneal transparency [20-25]. To determine the distribution of keratocan in the mouse cornea, the whole mount electro-immunostaining of wild type and keratocan null *Kera^−/−^* (keratocan knockout) mouse corneas was performed using goat anti-keratocan IgG Alexa 555 and normal non-immune goat IgG Alexa 555 conjugates. After electrophoresis, confocal 3D pictures revealed that goat anti-keratocan Alexa 555 conjugates preferentially reacted to corneal stroma but not to the corneal epithelium or endothelium. The labeling displayed a regular and characteristic striped pattern seen with conventional immunostaining in mouse corneal sections (Figure 2A). No signal was found in the corneas of keratocan null *Kera^−/−^* mice with anti-keratocan conjugates or in the corneas of wild type mice stained with non-immune normal goat IgG (Figure 2B,C). In contrast, the results using conventional whole mount fluorescent immunostaining with anti-keratocan-Alexa 555 antibody showed that the antibody couldn’t enter the corneal stroma and that large amounts of non-specific binding were accumulated on the corneal epithelium and endothelium (Figure 2D).

### Whole mount electro-immunofluorescent staining of intracellular structural protein

**Figure 3:**
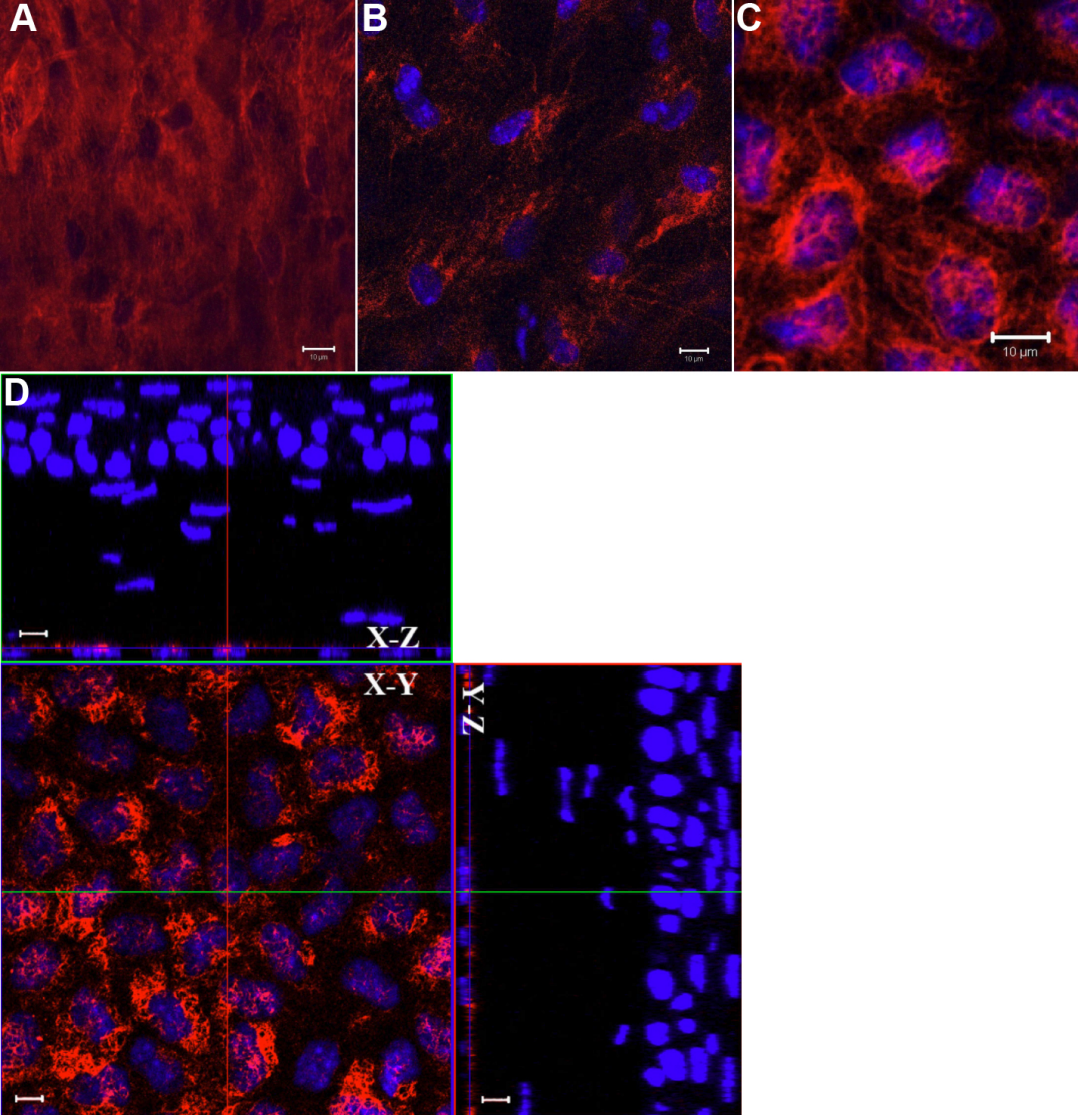
Distribution of β-tubulin in mouse cornea. β-Tubulin (red) exhibits an extensive arrangement in the epithelium, and the de-convoluted cytoskeleton fills the whole cell body (**A**). In the corneal stromal cells, the microtubule extended from the nucleus to the cell membrane (**B**). In the corneal endothelium, the convoluted microtubules twine around the nuclei, and very few microtubules extended to cell membrane (**C**). Following the conventional whole mount immunostaining, only the corneal endothelium was labeled by anti-β-tubulin (**D**). Scale bar: 10 μm.

Tubulin is an intracellular structural protein, which is frequently applied to studies of cytoskeleton [26-28]. To visualize the distribution of β-tubulin in the mouse corneal cells, mouse anti-β-tubulin monoclonal antibody conjugated with Alexa 555 was electrophoresed into wild type mouse corneas. CLSM showed that the intracellular microtube in superficial epithelial cells and stromal cells fill the whole cell body. However, in wing cell layers of the epithelium and endothelium, β-tubulin was distributed around the nuclei (Figure 3A–C). However, after conventional whole mount immunostaining, only the corneal endothelium was labeled by anti-β-tubulin antibody (Figure 3D).

**Figure 4:**
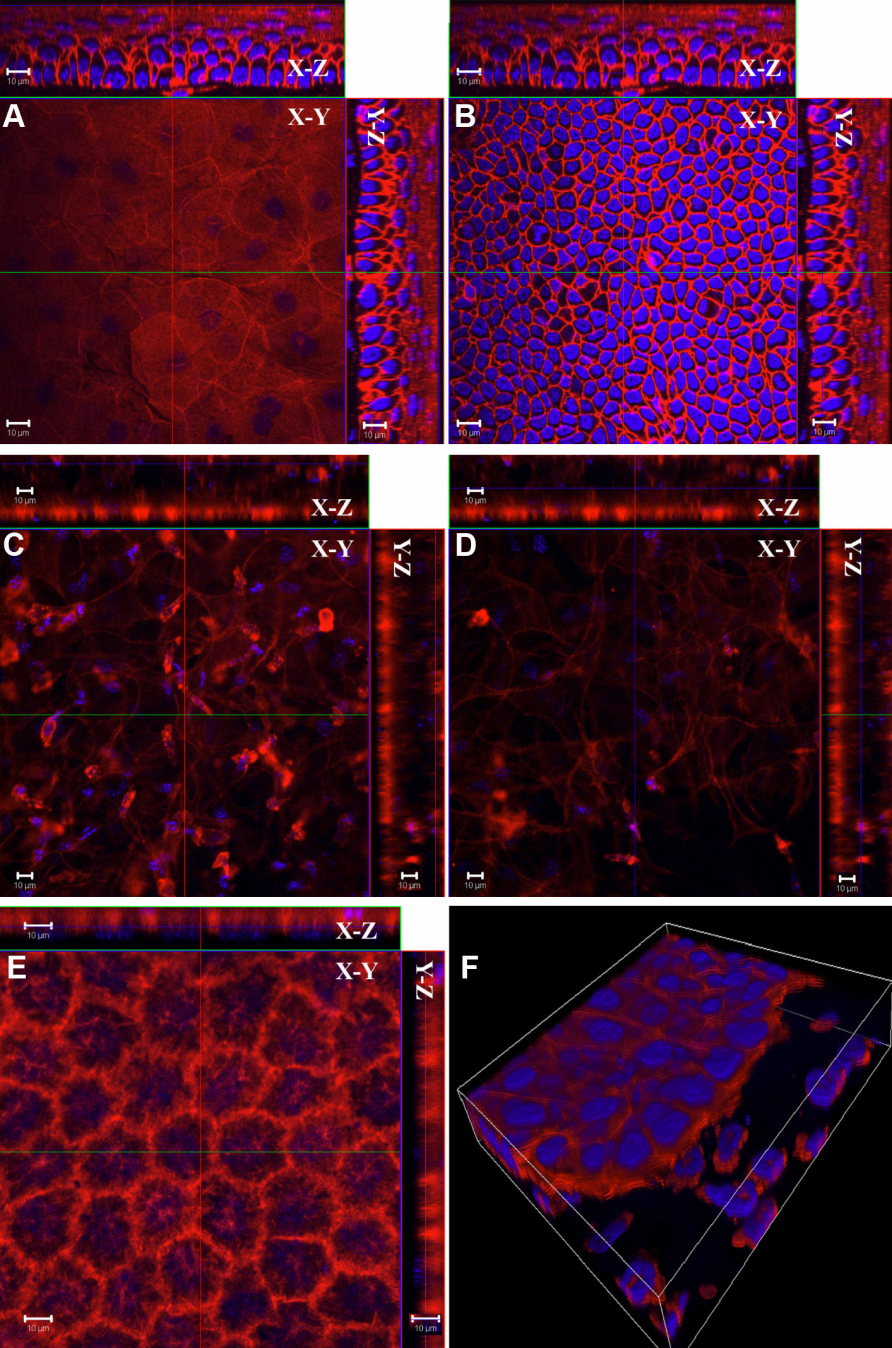
Distribution of F-actin in the mouse corneal cells and formation of filopodia and lamellipodia during healing of epithelium debridement. In the superficial epithelium, F-actin (red) is homogeneously distributed within whole cell body (**A**). However, in the corneal basal and wing cell layer, the microfilament networks are mostly gathered in the cytoplasmic circumference (**B**). In the corneal stroma, phalloidin labeling displays large and flat dendritic cell body of keratocyte (**C**,**D**). In the corneal endothelium, the distribution of F-actin gathered in the circumferential cytoplasm similar to that of the basal epithelial cell layer (**E**).The squamous epithelial cells at the leading edge altered the polygonal cell shape into spindle-shape and scallop-shape that oriented onto the substratum with scallop-shaped lamellipodia and filopodia. Polymorphonuclear neutrophils invading the stroma following corneal epithelium debridement were readily visible (**F**). Scale bar: 10 μm.

**Figure 5:**
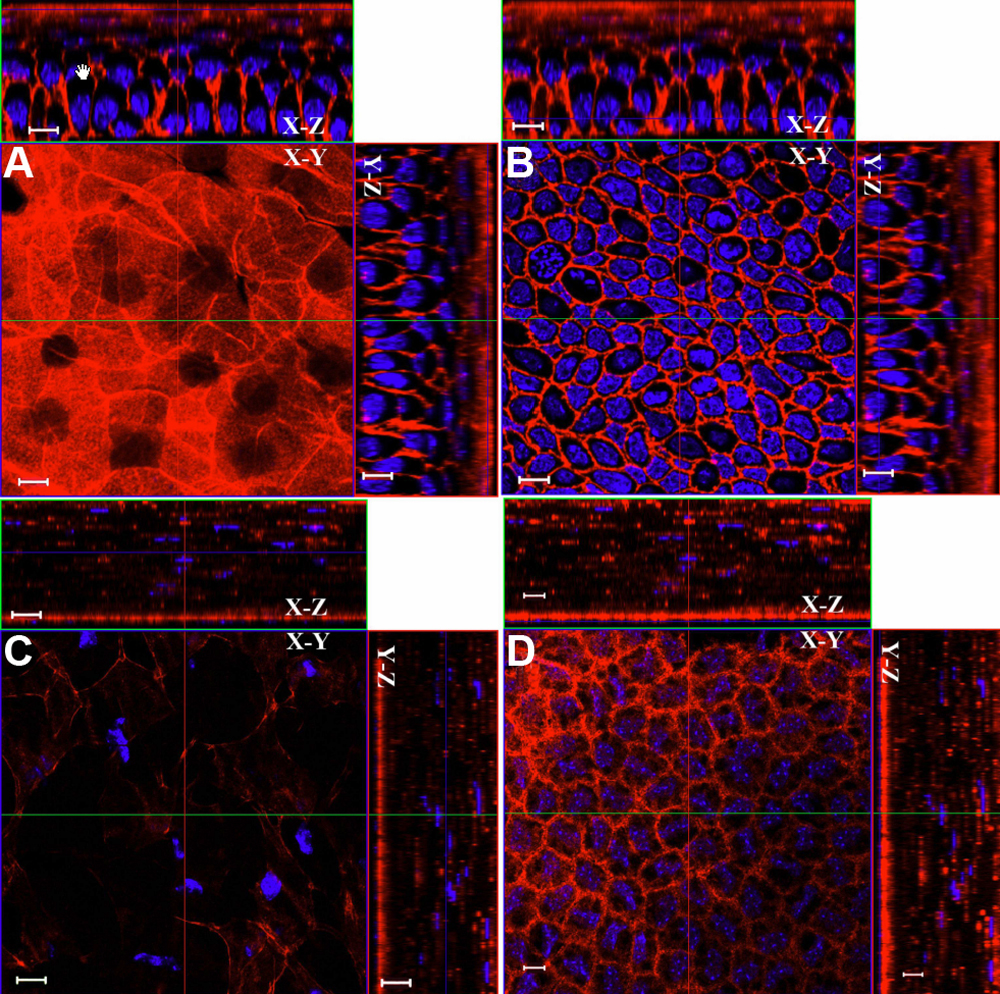
Distribution of F-actin in the mouse corneal cells after the conventional whole mount staining of phalloidin. In the superficial epithelium, F-actin (red) is homogeneously distributed in the whole cell body (**A**). In the corneal basal and wing cell layer, the microfilament networks are mostly gathered in the cytoplasmic circumference (**B**). In the corneal stroma, phalloidin labeling displays large and flat dendritic cell body of keratocyte (**C**). In the corneal endothelium, the distribution of F-actin gathered in the circumferential cytoplasm similar to that of the basal epithelial cell layer (**D**). Scale bar: 10 μm.

Phalloidin binds F-actin and has been used as a convenient tool to study the cellular locomotion associated with changes in the distribution of F-actin stress fibers in normal and diseased tissues [29-32]. To determine the distribution of F-actin in mouse cornea, wild type mouse corneas were fixed in the mixture of 4% paraformaldehyde and 0.2% glutaraldehyde as described in Methods. Following the whole mount electrostaining with phalloidin-rhodamine, confocal microscopic examinations revealed that F-actin was widely distributed in all three cell layers of normal mouse corneas, i.e., epithelium, stroma, and endothelium. In the epithelium, labeling was homogeneously distributed in the cell body of superficial epithelial cells (Figure 4A). However, in the wing and basal cell layers, these microfilament networks were mostly gathered in the cell circumference within the cytoplasm (Figure 4B). In the corneal stroma, the population of very large, flat stellate, net-shaped cells were labeled (Figure 4C,D). In the endothelium, the microfilaments were distributed mostly in the circumferential cytoplasm like those in basal corneal epithelial cells (Figure 4E). These results are similar to that using conventional whole mount fluorescent staining (Figure 5A–D).

Cell migration is an important event in embryonic development and pathogenesis, e.g., tumor metastasis and wound healing [33-35]. To examine the feasibility of using whole mount electrostaining to evaluate wound healing following corneal epithelial debridement, whole mount electrostaining of cornea was performed 12–18 h after a corneal epithelial wound with the phalloidin-rhodamine conjugate. Confocal examination revealed that surface squamous epithelial cells adjacent to the wound border changed their polygonal cell shape into spindle-shape or scallop-shape that oriented along the direction of the wound margin. Some cells at the leading edge of the migrating epithelium extended their lamellipodia and/or filopodia onto the substratum to form the cytoplasmic protrusion of the scallop-shape or rectangle scaffold at epithelial leading edge (Figure 4F).

### Whole mount electro-immunofluorescent staining for integral membrane protein

**Figure 6:**
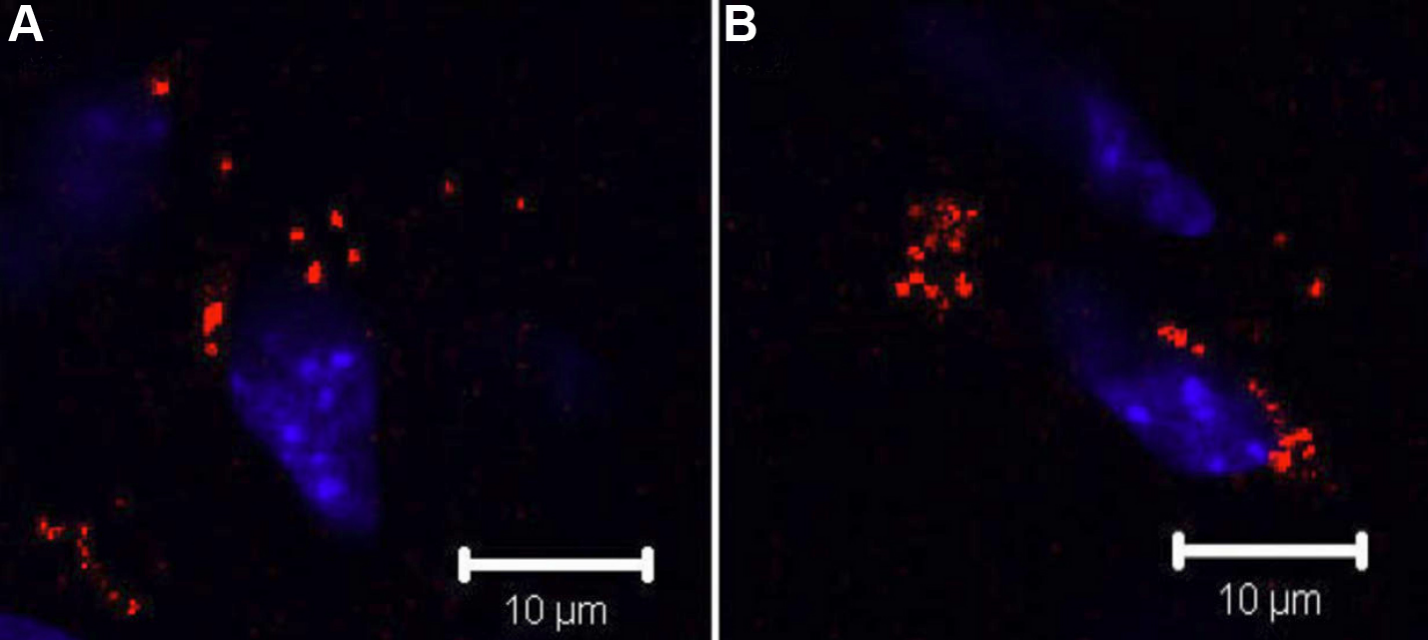
Distribution of FAK in mouse cornea after keratectomy. Keratectomy was performed in the center of corneas of wild type mice and healed for 3 weeks. The excised corneas were subjected to whole mount electroimmunostaining as described in Methods. CLSM revealed that anti-FAK Alexa 555 conjugate specifically labeled the cytoplasmic membrane of stromal cells three weeks after keratectomy. FAK is shown in red. Scale bar: 10 μm

**Figure 7:**
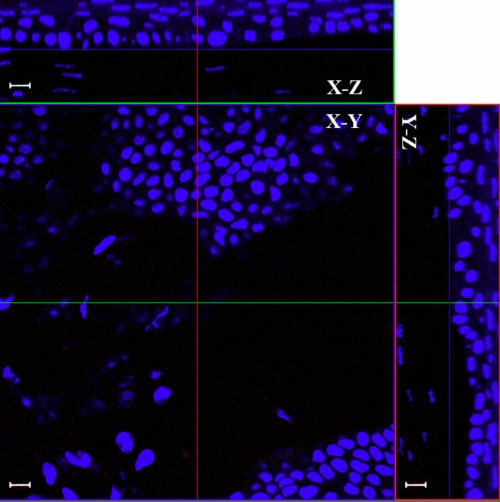
Conventional whole immunostaining with anti-FAK antibody. Three weeks after keratectomy, no signals were found in the corneal stroma through the conventional whole mount fluorescent immunostaining with anti-FAK-Alexa 555. Scale bar: 10 μm.

To examine the efficacy of electro-immunostaining on the detection of integral membrane proteins, especially in deep tissues, we determined the expression pattern of focal adhesion kinase (FAK) in mouse corneal stroma after corneal keratectomy. FAK transduces the signal of integrin binding to extracellular matrix and is found in the cell membrane where the cytoskeleton interacts with proteins of the ECM [36]. FAK is involved in the progression of tumor metastasis and wound healing [37-39]. Keratectomy was performed in C57BL/6J mice. After three weeks, the corneas were harvested and fixed in a mixture of 4% paraformaldehyde and 0.2% glutaraldehyde and then electrophoresed with mouse anti-FAK monoclonal antibody conjugated with Alexa 555. The results of CLSM showed that FAK labeling was specifically distributed in the injured corneal stromal area (Figure 6), and not in the unwounded region or in the corneal epithelium (data not shown). In contrast, anti-FAK-Alexa555 conjugates fail to label the mouse cornea using conventional whole mount fluorescent immunostaining (Figure 7).

**Figure 8:**
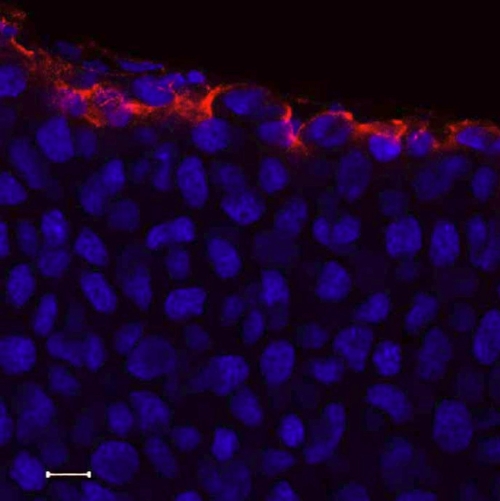
Conventional immunostaining protocol with short-term fixation. Short-term fixation didn’t improve the penetration of anti-β-tubulin Alexa 555 into normal mouse cornea. CLSM revealed that only cells near the cutting margin of the cornea were stained by anti-β-tubulin Alexa 555. Scale bar: 10 µm.

**Figure 9:**
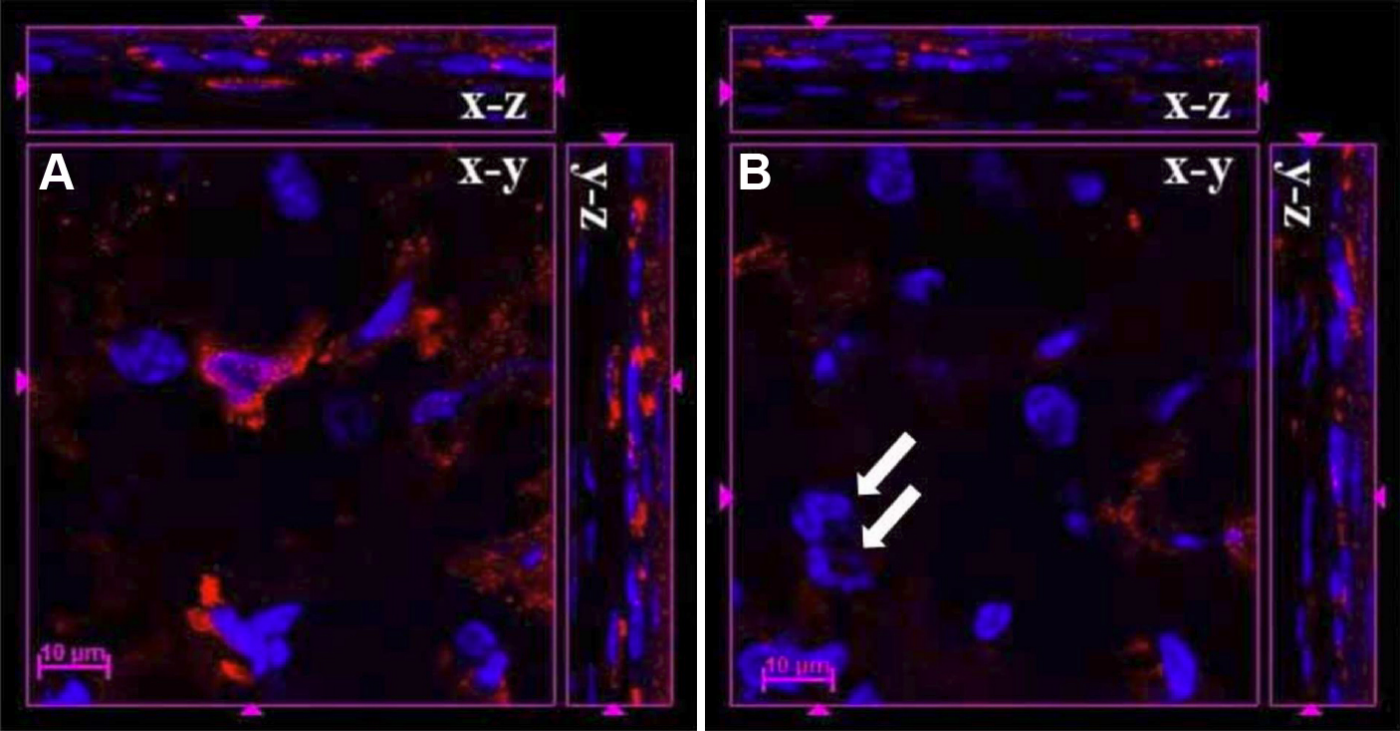
Inflammatory condition improved the penetration of anti-CD45 antibody in the mouse cornea. Leukocytes in the corneal stroma were labeled by rat anti-mouse CD45 antibody (red) 24 h after intrastromal LPS injection, and the signals were stronger on the upper side near the cutting edge than that on the down side. Inflammation caused by LPS improved the penetration of the anti-CD45 antibody that labeled leukocytes in the corneal stroma, but CD45 labeling was limited in the corneal epithelium and anterior stroma. The images of Z-stack clearly showed that leukocytes located in the epithelium and anterior stroma were labeled by CD45 antibody as shown in the XZ and YZ planes of the cut view of Z-stack CLSM images. In **A **the image of photo-section 29 is shown. In **B **the image at photo-section 32 (3 µm deeper from photo-section 29) is shown. White arrows show leukocytes that were not labeled by anti-CD45 antibody. Scale bar: 10 µm.

It has been reported that short-term fixation can improve the penetration of the staining reagents [40,41]. To verify the feasibility for different reagents, we fixed the cornea in 4% paraformaldehyde for 30 min and stained with mouse anti-β-tubulin-Alexa 555. To test this procedure on inflammatory cornea, we used the same procedure to stain the cornea 24 h after intrastromal injection of lipopolysaccharide (LPS) with rat anti-mouse CD45 antibody. The results clearly showed that only cells near the cutting margin were labeled by the anti-β-tubulin antibody (Figure 8). The inflammatory condition caused by LPS improved the penetration of the anti-CD45 antibody and allowed leukocytes to be labeled in the anterior corneal stroma by the antibody (Figure 9), but CD45 labeling was limited in the corneal epithelium and anterior stroma. Analysis of Z-stack images clearly showed that leukocytes located in the epithelium and anterior stroma were labeled by CD45 antibody as shown in the YZ and XZ planes of Figure 9A,B. Leukocytes labeled by anti-CD45 can be seen in the XY plane of photo-section 29 (Figure 9A). It is of interest to note that the antibody failed to label leukocytes located just 3 µm deeper in the XY plane of photo-section 32 (Figure 9B).

## Discussion

In this report, we describe the development of a novel technique for whole mount electro-immunofluorescent staining. This technique has several advantages: (1) enhanced permeability of the staining reagents, e.g., IgG conjugates by electrophoresis; (2) reduction of non-specific binding by removal of unbound IgG molecules via the electric current; and (3) efficient immunostaining completed overnight.

Conventional whole mount immunostaining is usually based on protocols used in regular immunohistochemical designs of tissues sections (paraffin and cryosections) [10,12,13,42,43]. The major obstacles of staining compact and dense tissues by immunohistochemistry derive from the poor penetration of staining reagents and non-specific binding [9,10,12,13,15]. In fact, these pitfalls mutually aggravate each other. Non-specific binding and high background can be partly reduced via enhanced penetration by changing fixatives used, e.g., picric acid/formaldehyde and methanol/formalin mixtures [10,12,15]. However, the processes of dehydration, clearing, and rehydration might extract antigens from tissues. Therefore, the conventional tissue processes reduced the sensitivity of detecting smaller amounts of antigens in tissues. Nonetheless, non-specific binding and high background often cannot be easily resolved using these staining procedures. Nonionic detergents, such as Triton X-100, NP-40, etc., have been used in immunostaining to increase the permeability of the cell and organelle membranes for staining reagents into tissues and to lower the background. It has been suggested that immunoreagents could penetrate through 100 μm into tissue sections [18,19]. However, the results of later studies using serial tissue sections of such immunohistochemical procedures indicated that penetration of immunoreagents was actually restricted to the superficial 8–9 µm [9].

Corneal stroma structurally shares a similar nature with neural tissues that demonstrate poor permeability for antibody conjugates to reach deep into tissues. The corneal epithelium and endothelium function as physical barriers that prevent antibody conjugate to freely penetrate into the tissue due to the presence of intercellular tight junctions of superficial corneal epithelial and endothelial cells [44,45]. Removal of the epithelium and/or tight junction, ZO1 (zona occludens) complex of the endothelium allows the penetration of protein molecules as large as 60–90 kDa into the corneal stroma [46,47]. The molecular weight of IgG used in the present study is about 150 kDa, which can readily penetrate into all three different corneal cell layers, i.e., epithelium, stroma, and endothelium.

Other problems in whole mount immunohistochemistry are non-specific binding and high background. They usually result from extended incubation time and high concentrations of the staining reagents to allow better penetration of antibodies into tissues. We used about 0.1–1 μg of antibodies (at about 1–10 μg/ml) per sample in our whole mount staining protocols, a concentration comparable to that of conventional immunostaining with cryo- and paraffin sections. The antibody conjugates driven by electric current bind to specific antigen epitopes present in deep tissue layers while unbound IgG molecules and/or those of non-specific low binding affinity IgG molecules continue to move in the electric field and move out of the tissues. Thus, it yields low background and reduces non-specific reactions. The quality of experimental outcome depends on the affinity of IgG molecules to antigens. Therefore, as one would expect, high affinity antibodies yield better results than those having low affinity.

Cell migration is essential for numerous biology processes such as wound healing, embryonic development, inflammation, and tumor cell metastasis. The basic mechanism that accounts for cell migration along the substratum is polarized actin polymerization and the formation of focal adhesion complexes. The filopodia and/or lamellipodia at the leading edge of migrating cells consist of actin filaments. These cell protrusions interact with the extracellular matrix via cell surface receptors such as integrin and promote cell locomotion [31,32]. Using regular whole mount immunostaining, these thin filopodia and lamellipodia, as well as adherence complexes, are often complicated by high background. The whole mount electrostaining readily recognized filopodia and lamellipodia at the leading edge of epithelium debridement by CLSM.

The method described herein displays a reliable and useful technique of whole mount immunostaining. It especially enables the improvement of the staining reagent penetration into the tissues by electric force. The unbound antibody or non-specific binding was simply removed, and the background was decreased. On the other hand, this technique depends on the mobility of the staining reagent in the buffer. So, the staining reagents that have the same isoelectric point as the buffer pH cannot be used in our whole mount electro-immunostaining. In theory, it is possible to perform double even triple immune fluorescence staining. However, we have not tried such tasks in our studies. Nevertheless, the success of double and triple immunostaining relies on the isoelectric points and binding affinities of antibodies employed against various antigens. Ideally, these antibodies should share similar and/or identical isoelectric points and similar high binding affinity to different antigens.
